# Things Worth Remembering

**Published:** 1894-01

**Authors:** 


### THINGS WORTH REMEMBERING.

It is authoritatively stated that headache almost always yields to the simultaneous application of hot water to the feet and back of the neck.

Ordinarily one woman in eight is sterile, but among women who have fibroids one in three is sterile. (Parvin.)

In facial erysipelas, where you cannot conveniently apply ordinary means, paint the part with ten per cent. iodoform collodion. (Prof. Gross.)

In posterior displacement of the uterus, always replace the organ before introducing the pessary; the frequent failure of its use is generally due to this cause. (Parvin.)

Where there is a collection of foreign matter, as pus, in the antrum of highmore, extract the first molar tooth (or more, if necessary), and drain the cavity in this way. (Sajous.)

For specific vaginitis, Prof. Parvin ordered mucilaginous injections and warm hip-baths in the acute stage, followed by injections of 1:100 corrosive solutions and tampons of boracic acid and glycerine.

Gelsemium will often do more good in irritable bladder than any other remedy. It is especially adapted to those women of hysterical type troubled by irritability at the neck of the bladder, calling for constant urination.

Without exception, the first symptom of pregnancy is an increased frequency of the desire to micturate.

Rhus aromatica, or the fragrant sumach, which grows all through the Northern States, is strongly recommended for incontinence of urine in atonic states of the bladder. From ten to fifteen drops of the tincture are given three times a day.

Salicylic acid is highly recommended as an application to ring-worm. It may used as an ointment, but is much better as a saturated solution in collodion. One application is often all that is necessary to effect a cure, but it may be repeated if necessary. The pain caused is not usually severe.

Boro-tartrate of potassium is the first remedy for calculus in pelvis of kindey; a weak solution must be used, and for a long time, a strong being detrimental. (Bartholow.)

Drop into urine in a test tube a few drops of the tincture of guaiac, heat it about 100°, and if it turns pale blue, pus is present in the urine.

Houghton, of Dublin, says that two hours of severe mental labor abstracts as much vital strength from the system as a whole day of physical labor.

Unna treats "red nose" with zinc-and-sulphur ointment externally and ichthyol internally.—*Massachusetts Medical Journal. Canada Lancet.*

